# Neuroactive Steroids and GABAergic Involvement in the Neuroendocrine Dysfunction Associated With Major Depressive Disorder and Postpartum Depression

**DOI:** 10.3389/fncel.2019.00083

**Published:** 2019-03-08

**Authors:** Jamie Maguire

**Affiliations:** Neuroscience Department, Tufts University School of Medicine, Boston, MA, United States

**Keywords:** major depression, postpartum depression, stress, HPA axis, neurosteroids, GABA

## Abstract

Stress and previous adverse life events are well-established risk factors for depression. Further, neuroendocrine disruptions are associated with both major depressive disorder (MDD) and postpartum depression (PPD). However, the mechanisms whereby stress contributes to the underlying neurobiology of depression remains poorly understood. The hypothalamic-pituitary-adrenal (HPA) axis, which mediates the body’s neuroendocrine response to stress, is tightly controlled by GABAergic signaling and there is accumulating evidence that GABAergic dysfunction contributes to the impact of stress on depression. GABAergic signaling plays a critical role in the neurobiological effects of stress, not only by tightly controlling the activity of the HPA axis, but also mediating stress effects in stress-related brain regions. Deficits in neuroactive steroids and neurosteroids, some of which are positive allosteric modulators of GABA_A_ receptors (GABA_A_Rs), such as allopregnanolone and THDOC, have also been implicated in MDD and PPD, further supporting a role for GABAergic signaling in depression. Alterations in neurosteroid levels and GABAergic signaling are implicated as potential contributing factors to neuroendocrine dysfunction and vulnerability to MDD and PPD. Further, potential novel treatment strategies targeting these proposed underlying neurobiological mechanisms are discussed. The evidence summarized in the current review supports the notion that MDD and PPD are stress-related psychiatric disorders involving neurosteroids and GABAergic dysfunction.

## Introduction

Here we review evidence supporting a role for GABAergic dysfunction, altered neurosteroid signaling, stress, and HPA axis dysregulation in both MDD and PPD. This review will focus solely on MDD and PPD. Although there is evidence for a role for GABA, neurosteroids, and the HPA axis in premenstrual dysphoric disorder, this topic has been nicely reviewed previously (Girdler and Klatzkin, 2007). Further, we provide evidence for an interaction between these proposed mechanisms in the underlying neurobiology of depression, in which stress can induce HPA axis dysfunction and altered neurosteroid signaling which can impact GABAergic inhibition in depression-relevant circuits. Conversely, GABAergic dysfunction can induce dysregulation of the HPA axis, altered stress reactivity, and neurosteroid signaling which may also cause dysfunction in depression-relevant circuits. We propose that depression involves a “state change” similar to what has previously been proposed (Schiller et al., 2014). Further, dysregulation of neurosteroids and/or HPA axis dysfunction may play a role in affective switching (Schiller et al., 2014), although this has yet to be thoroughly explored. This hypothesis also suggests that treatments which restore the healthy state may be capable of prolonged therapeutic effects, such as what has been shown in recent clinical trials with a synthetic neurosteroid, brexanolone (Kanes S. et al., 2017; Kanes S.J. et al., 2017). Future studies are required to determine the role of GABAergic dysfunction, altered neurosteroid signaling, stress, and HPA axis dysregulation in the state changes associated with both MDD and PPD.

This is a vast topic and therefore it is impossible to comprehensively review all of the points regarding GABAergic signaling, neurosteroids, and stress in depression. Instead, we attempt to provide a macroscopic view, examining the common threads linking GABAergic signaling, neurosteroids, stress, and depression and direct the reader to available resources with a more focused approach to subtopics covered in this review. Furthermore, the majority of the studies highlighted in this review have employed only male subjects. Therefore, it remains unclear whether these studies translate to female subjects. Given what we know about sex differences in stress reactivity (Bale and Epperson, 2015), behavior (Shansky, 2018), and gonadal hormone-linked changes in GABA_A_Rs (Maguire and Mody, 2009), it is likely that many of these findings may not translate to female subjects and it is imperative that we evaluate sex differences in these relationships.

## HPA Axis

The body’s neuroendocrine response to stress is mediated by the HPA axis. In response to a real or perceived stressor, corticotropin-releasing hormone (CRH) neurons in the PVN are activated and release CRH into the blood stream via the hypophyseal portal system. CRH stimulates the release of ACTH from the pituitary gland, which then triggers the release of corticosterone from the adrenal gland. In parallel, CRH neurons, in addition to other neuropeptide-containing neurons, including enkephalin, dynorphin, arginine-vasopressin, angiotensin, and oxytocin, project to the rostral ventrolateral medulla (RVLM) and initiate the autonomic response to stress. In tandem, these neuroendocrine responses to stress initiate the “fight or flight” response, coordinating the physiological and behavioral response to stress. In addition to their well-established role in governing HPA axis function, we now appreciate that CRH neurons in the PVN also have central projections which coordinate the behavioral response to stress (Fuzesi et al., 2016). Thus, CRH neurons in the PVN are critical mediators of stress reactivity and, as such, their activity is tightly regulated (for review see Herman et al., 2003; Larsen et al., 2003; Ulrich-Lai and Herman, 2009), predominantly by GABAergic signaling (for review see Decavel and van den Pol, 1990; Herman et al., 2004).

## GABAergic Regulation of the HPA Axis

Corticotropin-releasing hormone neurons, which govern the activity of the HPA axis, are tightly controlled by GABAergic signaling (Decavel and van den Pol, 1990, 1992) (for review see Herman et al., 2004; Cullinan et al., 2008). The importance of GABAergic regulation of CRH neurons is reflected in the fact that approximately 1/3 of the inputs onto these neurons are GABAergic (Miklos and Kovacs, 2002). Further, there is a high density of GABA inputs into the PVN, estimated to be at a density greater than 20 × 10^6^ synaptic contacts per mm^3^ (Miklos and Kovacs, 2002). Many regions regulating HPA axis function, including many cortical and limbic regions, involve an intermediate GABAergic neuron in a region surrounding the PVN, a region known as the peri-PVN (for review see Herman et al., 2004; Cullinan et al., 2008). Other GABAergic inputs into the PVN originate in the subparaventricular zone, the anterior hypothalamic area, dorsomedial hypothalamic nucleus, the medial preoptic area, lateral hypothalamic area, and from multiple nuclei within the bed nucleus of the stria terminalis (BNST; Cullinan et al., 1993; Roland and Sawchenko, 1993) (for review see Herman et al., 2004; Cullinan et al., 2008). Pharmacological manipulations have demonstrated that GABAergic inhibition plays a critical role in controlling the activity of the HPA axis at the level of the PVN (Cullinan et al., 2008; Marques de and Franci, 2008; Sarkar et al., 2011). For example, microinjection of bicuculline into the PVN increases the stress-induced corticosterone levels; whereas, microinjection of muscimol into the PVN reduces the stress-induced elevations in corticosterone (Cullinan et al., 2008). These data demonstrate a critical role for GABA in the regulation of the HPA axis at the level of the PVN and, therefore, suggest that GABA modulators, such as neurosteroids, may influence HPA axis function.

### Focus on Neurosteroids

Metabolites of steroid hormones, termed neuroactive steroids or neurosteroids, can rapidly modulate neuronal activity via non-genomic actions. Neuroactive steroids (NAS) are metabolites of steroid hormones, which independent of their site of origin, are capable of exerting effects on neural activity; whereas, the term “neurosteroids” refers specifically to steroid hormone metabolites which are synthesized locally in the brain either *de novo* or from peripherally derived precursors (Baulieu and Robel, 1990). Collectively, NAS and neurosteroids can act as positive allosteric modulators (PAMs) or negative allosteric modulators (NAMs) on a range of receptors, including both glutamate and GABA_A_ receptors (GABA_A_Rs) (Dubrovsky and Steroids, 2005). NAS and neurosteroids are known to exert potent anxiolytic and antidepressant effects (Dubrovsky and Steroids, 2005; Longone et al., 2008; Zorumski et al., 2013; Schüle et al., 2011) as well as anticonvulsant and sedative effects (Reddy, 2010). There are several classes of NAS, such as pregnane neurosteroids, which includes allopregnanolone and allotetrahydrodeoxycorticosterone (THDOC), androstane neurosteroids, such as androstanediol and etiocholanone, and sulfated neurosteroids, such as pregnenolone sulfate and dehydroepiandrosterone sulfate. For the purpose of the current review, we will focus on the pregnane neurosteroids, allopregnanolone and THDOC.

Central actions of allopregnanolone and THDOC are known to include actions on GABA_A_Rs (for review see Belelli and Lambert, 2005). GABA_A_Rs are heteropentameric receptors and the subunit composition dictates the subcellular localization, kinetics, and pharmacology of these receptors (Hevers and Luddens, 1998; Pirker et al., 2000; Kittler et al., 2002; Mody and Pearce, 2004). GABA_A_Rs incorporating the δ subunit are predominantly localized extrasynaptically and contribute to tonic GABAergic inhibition (for review see Farrant and Nusser, 2005). These specific subtypes of GABA_A_Rs have also been shown to be particularly sensitive to neurosteroid modulation (Mihalek et al., 1999; Belelli et al., 2002; Brown et al., 2002; Wohlfarth et al., 2002; Spigelman et al., 2003). However, the binding sites for neurosteroid-mediated allosteric modulation and direct receptor gating of GABA_A_Rs have been identified within the α subunit transmembrane domain and on the α/β interface, respectively, rather than involving the δ subunit (Hosie et al., 2006). Although not directly involved in neurosteroid binding, it has been suggested that the presence of the δ subunit contributes to neurosteroid potentiation via effects on the efficacy of potentiation (Hosie et al., 2009). Interestingly, these receptors have also been demonstrated to play a role in the regulation of the HPA axis (Sarkar et al., 2011).

Given the well-established role of GABAergic signaling in the regulation of the HPA axis, it is not surprising that neurosteroids have also been demonstrated to impact HPA axis function (for review see Wirth, 2011; Crowley and Girdler, 2014). For example, pretreatment with either allopregnanolone or THDOC decreases the neuroendocrine response to stress, decreasing the stress-induced increase in stress hormones, including ACTH and cortisol (Owens et al., 1992; Patchev et al., 1996). It is thought that increased levels of neurosteroids function to modulate HPA axis function (for review see Gunn et al., 2011). However, the impact of neurosteroids on HPA axis function differs across species and, therefore, may have differential effects on stress reactivity. Although neurosteroids likely have widespread effects on stress-sensitive circuits which can influence HPA axis function (reviewed previously Gunn et al., 2011), there is also evidence for direct effects of neurosteroids on CRH neurons in the PVN, which govern the activity of the HPA axis. For example, allopregnanolone has been shown to regulate the expression of CRH in the PVN (Patchev et al., 1994, 1996). CRH neurons at the apex of HPA axis function have been shown to be regulated by tonic GABAergic inhibition mediated by neurosteroid-sensitive, δ subunit-containing GABA_A_Rs and infusion of THDOC into the PVN decreases the stress-induced elevations in circulating corticosterone (Sarkar et al., 2011). These studies suggest that neurosteroids may act on δ subunit-containing GABA_A_Rs on CRH neurons in the PVN to directly control HPA axis function in addition to effects in other brain regions indirectly modulating HPA axis function.

It is important to note that the majority of these studies focus on circulating neurosteroids. However, we know that neurosteroids can be synthesized in the brain (Stoffel-Wagner, 2003; Barbaccia, 2004), but we still have little knowledge of the impact of local neurosteroidogenesis in the brain let alone the potential involvement in HPA axis function.

## Impact of Stress on GABAergic Regulation of the HPA Axis

It is well established that stress impacts HPA axis function, with some of the studies implicating changes in GABAergic signaling following both acute and chronic stress. The majority of these studies focus on chronic stress rather than acute stress. This section will review what is known about the impact of stress on GABAergic constraint of the HPA axis.

### Acute Stress

Although numerous studies have examined changes in GABAergic signaling following acute stress, few of these studies have focused on the hypothalamus or on the regulation of the HPA axis. Despite the limited exploration of this topic, altered GABAergic signaling in the hypothalamus has been demonstrated following acute stress. For example, increased GABA levels have been demonstrated in the hypothalamus following acute restraint stress (Yoneda et al., 1983); however, [^3^H]GABA and [3H]Ro-15-1788 binding is decreased in the hypothalamus following acute cold stress and acute defeat stress, respectively (Miller et al., 1987; Acosta et al., 1993). There is also evidence of functional alterations in GABAergic signaling, evident from an increase in the frequency of sIPSCs in the PVN following acute restraint (Inoue et al., 2013), shown to involve glucocorticoid receptor activation and retrograde opioid signaling (Wamsteeker Cusulin et al., 2013) and increased burst firing of GABAergic interneurons in the peri-PVN area (Shin et al., 2011). These data suggest that GABA signaling is altered in the hypothalamus following several acute stress paradigms.

GABA acting neurosteroids have also been shown to influence HPA axis activation following acute stress. Levels of both allopregnanolone and THDOC have been shown to increase following stress in animal models (Purdy et al., 1991; Barbaccia et al., 2001; Akk et al., 2007; for review see Paul and Purdy, 1992) in both the plasma and the brain (Barbaccia et al., 1998). Similarly, allopregnanolone levels have also been demonstrated to increase in humans in response to either CRH or ACTH stimulation (Genazzani et al., 1998) or in response to a stressful period, episode, or test (Girdler et al., 2001; Droogleever Fortuyn et al., 2004). Pretreatment of rats with allopregnanolone, allopregnanolone-THDOC, or progesterone attenuates stress-induced increases in plasma ACTH and cortisol (Owens et al., 1992; Patchev et al., 1996). Allopregnanolone treatment also decreased CRH mRNA expression in the PVN and CRH-induced anxiety-like behaviors (Patchev et al., 1994). Conversely, immunoneutralization of allopregnanolone using anti-allopregnanolone serum enhanced the acute stress (acute cold swim stress)-induced increase in circulating corticosterone (Guo et al., 1995). However, local infusion of THDOC into the PVN exacerbates that stress response (Sarkar et al., 2011). These findings suggest that the neurosteroids allopregnanolone and THDOC can exert effects on that activity of the HPA axis at the level of the PVN and likely in other brain regions which exert control over the HPA axis.

Although not a direct link to stress, corticosterone has been shown to regulate CRH neurons and therefore the activity of the HPA axis. Acute stress evokes an increase in corticosterone, which plays a well-established role in the negative feedback onto the HPA axis involving actions on glucocorticoid receptors. In addition to this classic negative feedback mechanism regulating HPA axis function, corticosterone has recently been shown to influence the GABAergic control of CRH neurons (Colmers and Bains, 2018). Corticosterone enhances tonic GABAergic inhibition on CRH neurons via upregulation of postsynaptic, presumably extrasynaptic, GABA_A_Rs (Colmers and Bains, 2018). These data suggest a novel negative feedback mechanism regulating HPA axis function involving actions of corticosterone on the GABAergic control of CRH neurons.

GABAergic inhibition tightly controls the activity of the HPA axis at the level of CRH neurons in the PVN (see section “GABAergic Regulation of the Hpa Axis”). However, this regulatory mechanism is complicated by metaplasticity at the level of the PVN (Bains, 2014). Despite this added complexity, it is clear that the GABAergic control of CRH neurons and, thus, the HPA axis becomes compromised following acute stress. The inhibitory control of the HPA axis requires low intracellular chloride levels in CRH neurons, so that when GABA binds to GABA_A_Rs, chloride flows into the cell, hyperpolarizes and, thus, inhibits CRH neurons. The low levels of intracellular chloride in neurons is achieved by the K^+^/Cl^-^ co-transporter, KCC2. The cell surface expression and function of KCC2 is regulated by phosphorylation at specific residues, with phosphorylation at the Ser940 residue enhancing expression and function (Lee et al., 2007). Following acute restraint stress, there is dephosphorylation of KCC2 at residue Ser940 and a reduction in KCC2 expression in the PVN (Sarkar et al., 2011), resulting in compromised GABAergic control of CRH neurons (Hewitt et al., 2009; Sarkar et al., 2011). Thus, it appears that chloride homeostasis plays a role in the stress-induced GABAergic regulation of the HPA axis.

We know surprisingly little about how CRH neurons are coordinated to facilitate the neuroendocrine stress response. We often think of CRH neurons in the PVN as a homogeneous population that responds in synchrony to a stressor. However, recent studies have uncovered the remarkable diversity in CRH neurons at the molecular level (Roman et al., 2017). CRH expressing neurons have been proposed to indicate a state switch, conferring functional competence, rather than an identifying marker (Roman et al., 2017). These findings demonstrate that CRH neurons in the PVN are molecularly diverse, expressing GABAergic, glutamatergic, or dopaminergic markers (Roman et al., 2017). Another recent study visualizes the recruitment of CRH neurons during an acute stressor of varying intensities. Using two-photon calcium imaging, the magnitude of the response of individual CRH neurons in zebrafish has been shown to increase with increased stressor intensity (vom Berg-Maurer et al., 2016). Further, there is an increase in the recruitment of CRH neurons with increased stressor intensity (vom Berg-Maurer et al., 2016). This is the first study demonstrating the stressor intensity-dependent coordination of CRH neuron activation in the hypothalamus. Future studies are required to determine the mechanisms regulating the coordination of CRH neurons in the face of varying stress intensities and the potential role of GABAergic signaling in this process. It also remains unclear whether similar mechanisms are in place following chronic stress.

### Chronic Stress

Numerous alterations in HPA axis function have been observed following chronic stress, including changes in the expression of stress-related neuropeptides and altered synaptic plasticity, leading to long-term changes in HPA axis function. This topic is thoroughly reviewed in Herman and Tasker (2016) and, therefore, this review will focus solely on the changes in the GABAergic regulation of the HPA axis following chronic stress.

Following chronic stress, there is an overall decrease in inhibitory synaptic transmission on neurons in the PVN, evident from a decrease in miniature and spontaneous inhibitory postsynaptic currents (mIPSCs, sIPSCs) (Verkuyl et al., 2004), decreased expression of enzymes required for GABAergic synthesis (Acosta et al., 1993; Montpied et al., 1993; Bowers et al., 1998), decreased GABA levels (Acosta et al., 1993), and altered GABA_A_R subunit expression (Cullinan and Wolfe, 2000; Verkuyl et al., 2004) (for review see Maguire, 2014). Altered GABA_A_R subunit expression is also inferred from changes in the binding of [3H]GABA and [3H]flunitrazepam following chronic stress (Acosta et al., 1993; Braestrup et al., 2003). The decreased frquency of sIPSCs observed following chronic stress (Verkuyl et al., 2004) can be prevented by adrenalectomy (Verkuyl and Joels, 2003) or mimicked with exogenous corticosterone treatment (Verkuyl et al., 2005), suggesting that these changes occur in response to the neuroendocrine changes associated with stress.

GABA acting neurosteroids have also been shown to influence the activity of the HPA axis following chronic stress. The expression of the rate-limiting enzyme involved in neurosteroidogenesis, 5α-reductase expression (Reddy, 2006; Agís-Balboa et al., 2007) and allopregnanolone and THDOC levels are decreased following chronic stress (Serra et al., 2000; Dong et al., 2001; Pinna et al., 2003; Serra et al., 2003; Sanna et al., 2004; Matsumoto et al., 2007). Decreased levels of these neurosteroids may disinhibit the activity of the HPA axis, given the evidence that a reduction in neurosteroid levels are associated with reduced negative feedback onto the HPA axis (Serra et al., 2006; Evans et al., 2012) and the stress derived neurosteroid, THDOC, has been demonstrated to alter HPA axis function at the level of the PVN (Sarkar et al., 2011).

There is also evidence of altered neurosteroid sensitivity associated with chronic stress in humans. Individuals reporting chronic stress exhibit an increased sensitivity to allopregnanolone-induced response in saccadic eye velocity (Bäckström et al., 2013). Further, there is also evidence that altered neurosteroid levels is associated with the negative impact of stress (Sundström Poromaa et al., 2003), which may play a role in mood disorders (Heim et al., 2001).

GABAergic constraint of the HPA axis is also compromised following chronic stress, similar to observations following acute stress. KCC2 is dephosphorylated and downregulated following chronic social defeat stress (Miller and Maguire, 2014). These observed changes presumably compromise the GABAergic control of CRH neurons, similar to what is observed following acute stress, leading to hyperactivation of the HPA axis and elevated circulating corticosterone levels (Miller and Maguire, 2014). Chronic early life stress has also been demonstrated to induce a shift in E_GABA_ in parvocellular neurons in the PVN (Gunn et al., 2013). Collectively, these data suggest that chronic stress alters the GABAergic control of parvocellular neurons in the PVN, compromising GABAergic control of the HPA axis, and leading to HPA axis dysfunction associated with chronic stress. There is accumulating evidence from multiple laboratories demonstrating a role for KCC2 in the regulation of the HPA axis and altered chloride homeostasis in stress-induced HPA axis dysfunction. Future studies are required to determine whether targeting KCC2 would be a useful therapeutic target for the treatment stress-related disorders.

## Major Depressive Disorder

The DSM-5 criterion for a diagnosis of major depression states that an individual must be experiencing five or more symptoms, including depressed mood, diminished interest or pleasure in activities, change in body weight (more than 5% in 1 month), insomnia, psychomotor agitation or retardation, fatigue or loss of energy, feelings of worthlessness or excessive or inappropriate guilt, decreased ability to concentrate, or recurrent thoughts of death or suicidal ideation. These criteria were established in an effort to standardize diagnosis and in this sense have great utility. However, even within this set of criteria, there is room for tremendous variability in symptom presentation for the diagnosis of major depressive disorder (MDD). We now appreciate that major depression is a heterogeneous disorders and that this definition may encapsulate numerous disorders with different underlying pathophysiology (Goldberg, 2011). Further, it is well established that adult women are twice as likely to suffer from major depression and even present with a different constellation of symptoms (Altemus et al., 2014). However, very few studies have focused on sex differences which may hold important information regarding the underlying neurobiology of MDD.

### Stress in Triggering MDD

There is a clear relationship between stress and depression, with extensive evidence pointing to a role for stress in triggering or worsening depression and the evidence of neuroendocrine abnormalities associated with MDD (for review see Hammen, 2005). However, the relationship between stress and depression is complex in that stress does not always lead to depression in individuals and depression can arise in the absence of prior life stress (Monroe and Reid, 2009). There appears to be a relationship between the severity and temporal association of stress to the onset of MDD symptoms. Both acute and chronic stress has been shown to be associated with MDD onset (Hammen et al., 2009). There is also a relationship between acute and chronic stress in MDD, with acute stress being more strongly associated with MDD in individuals with an increased history of chronic stress (Hammen, 2005; Hammen et al., 2009), suggesting that chronic stress may be an important predictor of depression risk.

Diagnosis of major depression traditionally followed that stress played a role in reactive depression, occurring in the presence of stress; whereas, endogenous depression resulting from underlying neurobiological factors was independent of an influence by stress. However, we now appreciate that stress can broadly influence depression. Severe life stress has been shown to contribute to the risk of MDD in individuals diagnosed with both endogenous and nonendogenous subtypes (Frank et al., 1994).

**FIGURE 1:**
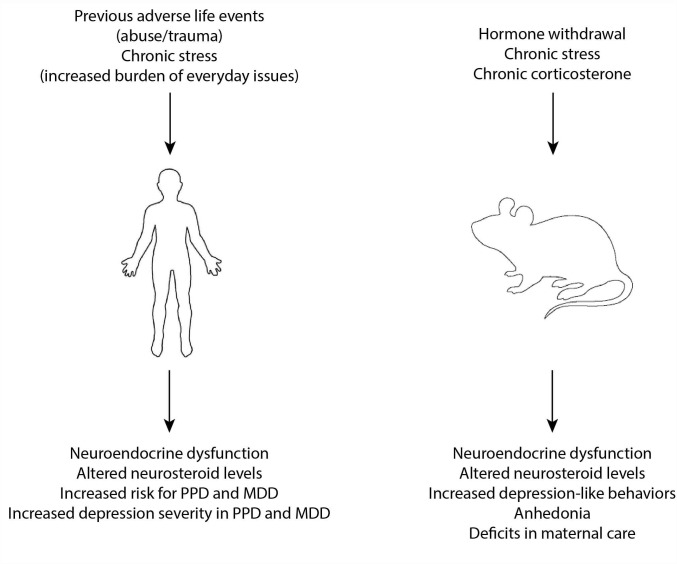
Risk factors and models of MDD and PPD. Previous adverse life events and chronic stress are known risk factors for the development of both MDD and PPD and have been associated with neuroendocrine dysfunction and altered neurosteroid levels which are thought to contribute to the underlying neurobiology of these disorders. In experimental models, hormone withdrawal, chronic stress, and chronic exposure to exogenous corticosterone have been used to model MDD and PPD and is associated with similar neuroendocrine disruption and altered neurosteroid levels.

Stressful life events have been demonstrated to be the strongest predictor of MDD (Kendler et al., 1993) (Figure 1). Stressors are also associated with severity of depression symptoms (for review see Tennant, 2002). Depression is a heterogeneous disorder, with variability in symptom presentation and likely the underlying neurobiology. An interesting study suggests that the nature of stress may correlate with different symptom manifestation, with death of loved ones or separation being associated with feelings of sadness, anhedonia, appetite loss; whereas, chronic stress is associated with fatigue and insomnia; whereas, the absence of adverse life events is associated with fatigue, increased appetite, and thoughts of self-harm (Keller et al., 2007). However, it remains unclear how stress contributes to the underlying neurobiology of depression, which remains a focus of research on MDD. Studies have implicated neuroinflammation, synaptic plasticity, and impact on stress-relevant networks as potential contributing factors to MDD (Slavich and Irwin, 2014; Gold et al., 2015; Richter-Levin and Xu, 2018). However, the mechanisms whereby stress precipitates MDD remain unclear and is worthy of further inquiry.

### Animal Models of MDD

Given the nature of symptoms in MDD, it is difficult to assess all the complex features of MDD. For example, assessing mood and suicidality are challenging in animal models. However, it is possible to assess other symptoms of MDD, such as helplessness, behavioral despair, anhedonia, and changes in sleep or appetite. Thus, behavioral assessments have largely focused on these features. Tests of learned helplessness, such as the forced swim test and tail suspension test, have been validated as measurements of depression-like behavior in rodents, given the effectiveness of antidepressant treatments in these tests (Vollmayr and Henn, 2001; Petit-Demouliere et al., 2005). Tests for anhedonia include intracranial self-stimulation (ICSS) and the sucrose preference test. Measurement of food consumption, circadian pattern of activity, and video-EEG assessment of sleep/wake states have also been used to assess features of depression-like behaviors.

The approaches discussed above are useful for assessing depression-like behaviors in rodents, but are not adequate for modeling MDD in rodents. Given the clinical evidence of a relationship between stress and depression summarized above, animal models for the study of MDD have largely focused on stress models or the administration of exogenous stress hormones (corticosterone) (Figure 1). Chronic corticosterone treatment is capable of modeling some of the behavioral and neurochemical changes associated with depression in adult rodents (Johnson et al., 2006; Zhao et al., 2008; Sterner and Kalynchuk, 2010), but, interestingly, not in adolescents (Waters and McCormick, 2011; Li et al., 2017). Chronic stress paradigms, including chronic unpredictable stress, chronic mild stress, or chronic social defeat stress have been utilized to induce depression-like behaviors in rodents (nicely reviewed in Krishnan and Nestler, 2011). It is well accepted that stress can be employed experimentally to model depression in rodents, with controllability, predictability, and chronicity/intermittency being important features (Anisman and Matheson, 2005). It is also appreciated that there is individual variability in behavioral and physiological response to stressors, in both human and experimental models, and attention should be paid to differences in resilient versus vulnerable animals to better understand the heterogeneity of depression. Further, it is also accepted that animal models are not entirely congruent with the human condition. Despite these recognized limitations, there are criteria which need to be fulfilled to validate animal models for the study of depression, including face validity modeling the symptoms of depression, predictive validity in responding to effective treatments, etiological validity modeling events which trigger or worsen depression (such as stress), and construct validity modeling similar neurochemical processes (Anisman and Matheson, 2005). However, there is a push toward incorporating different outcome measures, rather than solely focusing on behavioral assessments, for the study of MDD given that reliance on these tests has not produced new, useful therapeutic clinical options.

### Neuroendocrine Disruptions in MDD

Hyperexcitability of the HPA axis is a common feature of MDD (for review see Swaab et al., 2005; Pariante and Lightman, 2008; Figure 1). Patients with MDD exhibit increased baseline circulating cortisol levels and increased CRH in the brain and CSF (for review see Young et al., 2000). However, the mechanisms underlying HPA axis dysfunction and the contribution of these neuroendocrine abnormalities to the underlying neurobiology of MDD remain poorly understood. Hyperactivation of the HPA axis in MDD is thought to involve impairments in the negative feedback of glucocorticoids (for review see Pariante, 2006), which serve to limit HPA axis activation, and is supported by evidence of impairments in the dexamethasone suppression test (for review see Pariante, 2006). The field has struggled with whether these neuroendocrine abnormalities are a cause or a consequence of MDD. The evidence that exogenous corticosterone is sufficient to induce depression-like behaviors in rodents (Johnson et al., 2006; Zhao et al., 2008; Sterner and Kalynchuk, 2010) supports the notion that HPA axis abnormalities contribute to the underlying neurobiology of MDD. Further, evidence that previous adverse life events can cause HPA axis reprogramming resulting in neuroendocrine abnormalities reminiscent of those observed in MDD (for review see Pariante, 2006), lead us to believe that these risk factors induce neuroendocrine abnormalities which increase vulnerability to MDD rather than vice versa.

### Neurosteroids in HPA Axis Regulation in MDD

In addition to HPA axis hyperexcitability implicated in MDD, alterations in neurosteroids have also been suggested to play a role in the underlying neurobiology of MDD (for review see van Broekhoven and Verkes, 2003; Zorumski et al., 2013; Figure 1). Patients with MDD have decreased levels of allopregnanolone in the plasma and CSF (Romeo et al., 1998; Uzunova et al., 1998; Ströhle et al., 1999; Nappi et al., 2001) and there is a negative correlation between allopregnanolone levels and severity of depression symptoms (Uzunova et al., 1998; Nappi et al., 2001) (for review see Girdler and Klatzkin, 2007; Uzunova et al., 2006). Neurosteroids have been shown to regulate HPA axis function (for review see Gunn et al., 2011; Maguire, 2014) and it has been proposed that neurosteroid-mediated HPA axis dysfunction may contribute to depression (Crowley and Girdler, 2014). Experimentally, allopregnanolone levels are reduced in chronic stress models (Serra et al., 2000; Dong et al., 2001; Matsumoto et al., 2005; Serra et al., 2006), akin to those used to mode MDD. Expression of TSPO and 5α-reductase, the rate-limiting enzyme in neurosteroid synthesis, are decreased following chronic stress (Dong et al., 2001; Agís-Balboa et al., 2007; Rupprecht et al., 2010) and therefore may play a role in depression-like behaviors (for review see Zorumski et al., 2013). Consistent with this notion, finasteride treatment, which blocks 5α-reductase and therefore neurosteroidogenesis, leads to mood disorders including anxiety and depression, collectively referred to as post-finasteride syndrome, which is thought to involve altered neurosteroid levels (Melcangi et al., 2013). Based on these findings, targeting neurosteroidogenesis has been proposed to be a novel target for antidepressant treatment (Schüle et al., 2011). Treatment with allopregnanolone or treatments that increase allopregnanolone levels exerts antidepressant effects in animal models (Khisti and Chopde, 2000; Khisti et al., 2000; Frye and Walf, 2002) and decreases CRH expression in the PVN (Patchev et al., 1994) (for review see van Broekhoven and Verkes, 2003), suggesting that allopregnanolone can decrease the behavioral and neuroendocrine abnormalities associated with depression. Further evidence supporting a role for neurosteroids in the underlying neurobiology of MDD is the evidence that antidepressant treatment increases allopregnanolone levels on a timescale related to their antidepressant effects (Romeo et al., 1998; Uzunova et al., 1998; Ströhle et al., 1999; Strohle et al., 2002). These findings suggest involvement of GABA_A_R-targeting neurosteroids in MDD which has contributed to GABA being implicated in MDD, a topic discussed in the following section.

### GABAergic Hypothesis of MDD

A GABAergic deficit hypothesis of MDD has been proposed, implicating GABAergic dysfunction in the underlying neurobiology of MDD (Luscher et al., 2011). Indirect evidence for a role for GABA in MDD is based on the role of GABA in the regulation of the HPA axis, which has been implicated in MDD (summarized above in section “Stress in Triggering MDD” and “Neuroendocrine Disruptions in MDD”). Further, the antidepressant effects of neurosteroids, which act on GABA_A_Rs, also provides indirect evidence for GABA in MDD (reviewed in section “Neurosteroids in HPA Axis Regulation in MDD”, Romeo et al., 1998; Uzunova et al., 1998; Ströhle et al., 1999; Strohle et al., 2002). Direct evidence for a role for GABA deficits in MDD is based on decreased levels of GABA observed in patients with MDD, there is decreased expression of GABA synthesizing enzymes, altered expression of GABA_A_R subunits, and a reduction in the number of GABAergic interneurons in patients with MDD (nicely reviewed in Luscher et al., 2011; Zorumski et al., 2013). GABAergic deficits in MDD are also supported by evidence of polymorphisms in genes encoding for GABA_A_R subunits, including α1, α4, α5, α6, β1, β3, γ2, and δ (Luscher et al., 2011). Experimentally, altered GABA_A_R subunit expression can influence depression-like behaviors, but the direction and extent depend upon the specific GABA_A_R subtypes. For example, mice lacking the α3 subunit (α3^-/-^ mice) exhibit increased struggling and decreased immobility in the forced swim test, indicative of antidepressant-like effects (Fiorelli et al., 2008). In contrast, mice lacking the α2 subunit (α2^-/-^ mice) exhibit increased immobility in the forced swim and tail suspension tests, suggesting that the loss of the α2 subunit increases depression-like behaviors and it has been suggested that these receptors may play a critical role in mediating antidepressant actions (Fiorelli et al., 2008). The most convincing experimental evidence for GABAergic deficits in depression is the finding that a reduction in the expression of the GABA_A_R γ2 subunit in the forebrain (γ2^+/-^ mice) is sufficient to induce neuroendocrine abnormalities and anxiety-like and depression-like behaviors (Earnheart et al., 2007; Shen et al., 2010) reminiscent of MDD. Interestingly, only antidepressant treatments which are effective at restoring normal HPA axis function improves the depression-like phenotype of γ2^+/-^ mice (Shen et al., 2010). These data implicate GABAergic dysfunction in MDD and ties antidepressant treatment to restoration of neurosteroid levels and HPA axis function. However, it is important to note that GABAergic drugs, such as benzodiazepines, have not been shown to improve the core symptoms of depression, although they may be beneficial for treating anxiety or sleep disturbances associated with depression (Johnson, 1985; Birkenhager et al., 1995). Rather, emerging clinical studies suggest that targeting neurosteroids may be therapeutic and a better treatment option for MDD.

## Postpartum Depression

Postpartum depression (PPD) is classified in DSM-5 as “MDD, with peripartum onset.” Similar to major depression, diagnosis of PPD requires the presence of five or more of the following symptoms: depressed mood, diminished interest or pleasure in activities, change in body weight (more than 5% in 1 month), insomnia, psychomotor agitation or retardation, fatigue or loss of energy, feelings of worthlessness or excessive or inappropriate guilt, decreased ability to concentrate, or recurrent thoughts of death or suicidal ideation, and stipulates that symptom onset must occur during pregnancy or within the first 4 weeks following delivery. Along with other aspects of women’s heath, there have been a limited number of studies focused on PPD. We posit that investigations into the unique and common features of MDD and PPD will provide information about the underlying neurobiology of depression.

### Stress in Triggering PPD

The impact of stress on depression is well established as reviewed under Section “Stress in Triggering MDD”. There is also accumulating evidence that stress is a risk factor for PPD (for review see Swendsen and Mazure, 2000; Robertson et al., 2004; Figure 1). Ongoing stressors, such as lack of social support, marital issues, and socioeconomic status are all risk factors for PPD (for review see Robertson et al., 2004). In addition, previous adverse life events, such as childhood trauma or sexual abuse, have also been identified as risk factors for PPD (Meltzer-Brody et al., 2013; Guintivano et al., 2018; Meltzer-Brody et al., 2018). Stressful events occurring during the postpartum period have the strongest association with PPD (Paykel et al., 1980; O’Hara et al., 1984). An indication of this association is the evidence that women exhibiting PPD report significantly more life stress than non-depressed new mothers (O’Hara, 1986). Despite differences in methodology conducted in many studies examining the relationship between stress and PPD, there is overwhelming evidence of an association (for a nice review with attention to methodology, see Swendsen and Mazure, 2000). In addition to the role stress plays as a risk factor for PPD, stress (both current chronic stress as well as previous adverse life events) also impacts the severity of symptoms in PPD (see Swendsen and Mazure, 2000). Thus, although there are unique features of PPD and MDD, there are also some commonalities. For example, it appears that stress is a risk factor and can worsen depression symptoms in both PPD and MDD. However, it remains unclear how stress contributes to the underlying neurobiology of PPD. To better understand the relationship between stress and PPD, animal models have been employed and findings from preclinical studies are summarized in the following sub-section.

### Animal Models of PPD

Animal models have been employed in an attempt to achieve a better understanding of the underlying neurobiology of PPD. Although it is undeniably difficult to model such a complex psychiatric disorder, existing models are largely based on known risk factors or observations from the clinic which can be modeled in animals. Existing models include pseudo-pregnancy models or hormone withdrawal models, corticosterone- or stress-based models (nicely reviewed in Perani and Slattery, 2014; Figure 1). Based on the onset of symptoms of PPD, occurring at a time of dramatic hormone fluctuations, hormone withdrawal in the pseudo-pregnancy model is sufficient to induce depression-like behavior in rats (Galea et al., 2001; Stoffel and Craft, 2004) as well as anhedonia (Green et al., 2009; Navarre et al., 2010). An abrupt rather than gradual decline in hormone levels has been shown to induce increased stress reactivity and precipitate abnormal behaviors (Doornbos et al., 2009). The behavioral deficits associated with hormone withdrawal may involve reductions in neurosteroid levels given the evidence that blocking neurosteroidogenesis with finasteride increases depression-like behaviors (Frye and Walf, 2004). These data implicate changes in gonadal hormones in precipitating mood disorders during the postpartum period. However, there is also evidence that stress hormones may play a role. Based on the evidence for stress and HPA axis dysfunction in PPD (reviewed in section “Stress in Triggering PPD”), models employing exogenous corticosterone administration or chronic stress have been utilized to model PPD (nicely reviewed in Perani and Slattery, 2014). Treatment with exogenous corticosterone during the postpartum period/lactation induces depression-like behaviors and deficits in maternal care (Brummelte and Galea, 2010; Maguire and Mody, 2016). Rodents subjected to repeated stress during pregnancy exhibit depression-like behaviors (Smith et al., 2004; O’Mahony et al., 2006; Maguire and Mody, 2016), deficits in maternal care (Maestripieri et al., 1991; Pardon et al., 2000; Smith et al., 2004; Kurata et al., 2009; Brummelte and Galea, 2010; Nephew and Bridges, 2011; Murgatroyd and Nephew, 2013; Maguire and Mody, 2016), and elevated levels of corticosterone (Misdrahi et al., 2005; Maguire and Mody, 2016; for review see Perani and Slattery, 2014). Pup separation is also used to model both HPA axis dysfunction, depression-like behaviors, and deficits in maternal care (Boccia et al., 2007; Pawluski et al., 2009; Maniam and Morris, 2010). Collectively, these preclinical findings suggest an association between stress, HPA axis dysfunction, and PPD-like behaviors.

A causal relationship between HPA axis dysfunction and PPD-like behaviors was explored using mouse models which exhibit hypercortisolism during the peripartum period. Mice which lack the GABA_A_R δ subunit (*Gabrd^-/-^* mice) exhibit depression-like behaviors restricted to the postpartum period and deficits in maternal care (Maguire and Mody, 2008), a finding which has been attributed to the disinhibition of CRH neurons (Sarkar et al., 2011) resulting in elevated corticosterone levels during the peripartum period (Maguire and Mody, 2016). To further investigate the role of HPA axis dysfunction in PPD, a mouse model was generated which lacks the K^+^/Cl^-^ co-transporter, KCC2, in CRH neurons (KCC2/Crh mice), which has been shown to play a critical role in the stress-induced activation of the HPA axis. KCC2/Crh mice exhibit the inability to suppress the stress-induced activation of the HPA axis during the peripartum period, leading to elevated levels of corticosterone, PPD-like behaviors, and deficits in maternal care (Melón et al., 2017). These findings provide further, direct support for HPA axis dysfunction in PPD and highlight the utility of animal models for studying the underlying neurobiology of PPD.

### Neuroendocrine Disruptions in PPD

The peripartum period is accompanied by remarkable changes in the levels of gonadal hormones and neurosteroids (Mastorakos and Ilias, 2000; Bloch et al., 2003; Mastorakos and Ilias, 2003), characterized by high levels of estrogen, progesterone, and allopregnanolone. In addition, there are also changes in the functioning of the HPA axis during the peripartum period, in which there are elevated levels of stress hormones during pregnancy (Nolten et al., 1980) with a marked reduction and HPA axis hypofunction during the postpartum period (Magiakou et al., 1996). These changes serve numerous functions which are necessary for the development and protection of the fetus as well as preparing the mother for the physiological challenges of motherhood. Disruption in the orchestration of these neuroendocrine changes or responsivity to these hormones can have disastrous effects, including but not limited to preterm birth, adverse fetal outcomes, fetal morbidity, maternal morbidity, and PPD (Bloch et al., 2003; Frise and Williamson, 2013). The well-established influence of hormones and hormone withdrawal on mood (Schiller et al., 2015), lead to the assumption that hormonal dysregulation underlies perinatal depression for review see Meltzer-Brody, 2011). However, the reports of changes in hormone levels in women with perinatal depression is inconsistent, which may in part be a reflection of this heterogenous patient population. A critical discovery made by Bloch et al. demonstrated that hormone withdrawal only induced depression symptoms in women with a history of PPD (Bloch et al., 2000), suggesting an underlying vulnerability to hormone fluctuations in this population.

As described above, there are also profound changes in HPA axis function during the peripartum period. Given the well-known role for stress and neuroendocrine changes in MDD (see section “Stress in Triggering MDD” and “Neuroendocrine Disruptions in MDD”), HPA axis dysfunction has also been implicated in PPD (Magiakou et al., 1996; Wisner and Stowe, 1997; Bloch et al., 2003, 2005; for review see Meltzer-Brody, 2011). Although there are been conflicting findings regarding absolute changes in stress hormone levels associated with PPD, an elegant study demonstrated an exaggerated cortisol and increased depression symptoms upon withdrawal of gonadal hormones only in women with a history of PPD (Bloch et al., 2000). These findings suggest that HPA axis dysfunction may not be the primary deficit in PPD, but nonetheless may contribute to the pathophysiological processes involved in PPD. It is worth mentioning here that there is an interaction between the hypothalamic-pituitary-gonadal (HPG) and HPA axes, with clear evidence that stress disrupts HPG function and well-established changes in HPA axis function related to the HPG axis (for review see Mastorakos et al., 2006; Camille Melón and Maguire, 2016). Therefore, it is likely that changes in either the HPG or the HPA could influence that activity of the other system.

Neurosteroids in particular have been shown to be involved in regulating HPA axis function (Sarkar et al., 2011; for review see Gunn et al., 2011). Thus, it is possible that altered neurosteroid levels may contribute to HPA axis dysfunction in PPD. However, similar to the findings with gonadal hormones, measurements in absolute levels of neurosteroids associated with PPD have be inconsistent. Allopregnanolone levels are decreased in postpartum women, but were not found to significantly differ in women with PPD (Epperson et al., 2006); however, other studies have shown decreased levels of allopregnanolone during late pregnancy is negatively correlated with depression symptoms postpartum and has been suggested to be a predicting factor for PPD (Nappi et al., 2001; Hellgren et al., 2014; Crowley et al., 2016; Osborne et al., 2017). Significant alterations in GABA and neurosteroid levels have been observed in patients at risk for PPD (Deligiannidis et al., 2013; Deligiannidis et al., 2016). Again, the conflicting findings in neurosteroid levels in women with PPD are likely a reflection in the heterogeneity of this population. In patients which do show changes in neurosteroid levels, it is likely to impact mood as allopregnanolone has been proposed to be involved in switching between affective states (Schiller et al., 2014). These data suggest that neuroendocrine abnormalities may contribute to the underlying neurobiology of PPD as well as MDD (summarized in section “Neuroendocrine Disruptions in MDD,” Figure 1). Despite the unique features of PPD and MDD, there are clear similarities as well. It is possible that there are converging mechanisms leading to similarities in symptom presentation, which may include altered sensitivity to gonadal hormones and/or disruption in HPA axis function.

### Evidence for GABAergic Dysfunction in PPD

Given the ability of steroid hormones and neurosteroids to regulate GABA_A_Rs (Abramian et al., 2014; Modgil et al., 2017), which occurs throughout the peripartum period (Licheri et al., 2015), it is not surprising that alterations in GABAergic signaling has been implicated in the underlying neurobiology of PPD. In women at risk for developing PPD, GABA levels have been shown to be lower during the peripartum period (Deligiannidis et al., 2016). Further, GABA levels are negatively correlated with depression scores (Deligiannidis et al., 2016). Although differences in allopregnanolone levels have not been consistently found associated with PPD (for review see Amin et al., 2006), it has been suggested that women with PPD may differ in their response to decreases in GABA and allopregnanolone levels during the postpartum period (for review see Amin et al., 2006). Consistent with this notion, experimentally, expression of the GABA_A_R δ subunit has been shown to be regulated throughout the peripartum period (Maguire and Mody, 2008; Maguire et al., 2009) and mice incapable of regulating these receptors (*Gabrd^-/-^* mice) exhibit abnormal postpartum behaviors (Maguire and Mody, 2008). Steroid hormones and neurosteroids have been shown to regulate the expression and function of GABA_A_Rs (Mody and Maguire, 2011; Maguire, 2014). GABAergic signaling has been proposed to play a role in anxiolysis during the postpartum period; whereas, dysregulation in GABAergic signaling is thought to negatively impact mood during this period (Lonstein et al., 2014). Therefore, abnormalities in the ability of steroid hormones and neurosteroids to regulate GABA_A_Rs may represent a convergent mechanism between these factors associated with PPD and may be similar for MDD. Although the precise mechanisms and networks in which GABAergic signaling modulates mood remain unclear, it is evident that GABA, neurosteroids, and the HPA axis play critical roles in regulating mood.

## Commonalities Between MDD and PPD

The findings summarized in this review point to similarities between MDD and PPD. Despite the unique features of PPD, with obvious differences in temporal onset, it is also evident that MDD and PPD also have similar features, including symptom presentation, shared risk factors, and neuroendocrine disruptions. These data suggest that despite the fact that MDD and PPD are distinct disorders, there is evidence for similarities in the underlying neurobiology. Therefore, it is possible that there are convergent mechanisms which may be identified and targeted for treatment of both MDD and PPD.

## Novel Therapeutic Strategies for MDD and PPD

The known risk factors and biochemical changes identified in MDD and PPD suggest alternative targets for treatment. The evidence for HPA axis dysfunction in both MDD and PPD suggests that normalizing HPA axis function may be therapeutic for the treatment of these disorders. In fact, in a preclinical PPD model, suppressing the hyperactivation of the HPA axis during the peripartum period decreases depression-like behaviors and improves maternal care (Melón et al., 2017) and improves outcomes in MDD models (Khisti and Chopde, 2000; Khisti et al., 2000; Frye and Walf, 2002). However, we know rather little about how the HPA axis is regulated throughout the peripartum period and the mechanisms whereby the HPA axis becomes dysfunctional in MDD and PPD. Interestingly, neurosteroids have been implicated in regulation of the HPA axis during pregnancy and the postpartum period (Brunton and Russell, 2011; Brunton, 2015). It is tempting to speculate that decreased levels of neurosteroids associated with MDD and PPD may underlie HPA axis dysfunction and that neurosteroid-based treatments may be therapeutic in part by normalizing the HPA axis. Recently, neurosteroid-based treatments have been shown to be effective in decreasing depression scores in patients with MDD and PPD (Kanes S. et al., 2017; Kanes S.J. et al., 2017). Experimentally, a neurosteroid-based treatment similar to those employed clinically was shown to be effective at decreasing depression-like behaviors and restoring HPA axis function in preclinical PPD models (Melón et al., 2018). Thus, there is evidence that neurosteroid-based treatments are effective in both MDD and PPD and perhaps suggests that these compounds are targeting a similar underlying neurobiological mechanism, involving HPA axis dysfunction.

## Conclusion

This broad topic prevented a comprehensive review of each subtopic. Instead, the goal of this review was to present evidence suggesting that stress is a risk factor for both MDD and PPD, GABAergic dysfunction plays a role in both MDD and PPD, GABAergic signaling and neurosteroids regulate HPA axis function, and HPA axis dysfunction has been implicated in both MDD and PPD. Finally, there are similarities in MDD and PPD which despite the unique features of these disorders suggests that there may be convergence in the underlying neurobiology and, therefore, potential avenues for treatment which would be effective for both of these patient populations.

## Author Contributions

JM researched and synthesized the information and wrote the review.

## Conflict of Interest Statement

The author declares that the research was conducted in the absence of any commercial or financial relationships that could be construed as a potential conflict of interest.
